# SARS-CoV-2 spike protein-derived immunogenic peptides that are promiscuously presented by several HLA-class II molecules and their potential for inducing acquired immunity

**DOI:** 10.1016/j.heliyon.2023.e20192

**Published:** 2023-09-20

**Authors:** Yuki Yajima, Akemi Kosaka, Takayuki Ohkuri, Yoshihiko Hirohashi, Dongliang Li, Takeshi Nagasaki, Toshihiro Nagato, Toshihiko Torigoe, Hiroya Kobayashi

**Affiliations:** a Department of Oral and Maxillofacial Surgery, Asahikawa Medical University, Asahikawa, Hokkaido, Japan; b Department of Pathology, Asahikawa Medical University, Asahikawa, Hokkaido, Japan; c Department of Pathology, Sapporo Medical University School of Medicine, Sapporo, Japan; d Tsukuba Laboratory, Medical & Biological Laboratories Co., Ltd., Ina, Japan

**Keywords:** Helper T lymphocytes, Cellular immunity, T-cell monitoring, SARS-CoV-2

## Abstract

The current coronavirus disease 2019 (COVID-19) pandemic that is caused by the severe acute respiratory syndrome coronavirus 2 (SARS-CoV-2) has a significant threat to public health. Although vaccines based on the mRNA of the SARS-CoV-2 spike protein have been developed to induce both cellular and humoral immunity against SARS-CoV-2, there have been some concerns raised about their high cost, particularly in developing countries. In the present study, we aim to identify an immunogenic peptide in the SARS-CoV-2 spike protein to activate cellular immunity, particularly CD4^+^ helper T lymphocytes (Th cells), which are a commander of immune system. SARS-CoV-2 spike protein-derived peptides Spike_448-477_ and Spike_489-513(N501Y)_-specific CD4^+^ Th cell lines were generated by repetitive stimulation of healthy donor-derived CD4^+^T-cells with each peptide. Their HLA-restrictions were addressed by using blocking antibodies against HLA and HLA-transfected L-cells. The epitopes of Spike_448-477_-specific CD4^+^ Th cell lines were defined using a series of 7–14-mer overlapping truncated peptides and alanine-substituted epitope peptides. To address responsiveness of these CD4^+^ Th cell lines to several SARS-CoV-2 variants, we stimulated the CD4^+^ Th cell lines with mutated peptides. We addressed whether these identified peptides were useful for monitoring T-cell-based immune responses in vaccinated donors using the IFN-γ ELISpot assay. The Spike_448-477_ peptide was found to be a promiscuous peptide presented by HLA- DRB1*08:02, DR53, and DPB1*02:02. Although HLA-DPB1*02:02-restricted CD4^+^ Th cells did not response to some peptides with the L452R and L452Q mutations, the other CD4^+^ Th cells were not affected by any mutant peptides. We developed two tetramers to detect HLA-DRB1*08:02/Spike_449-463_- and Spike_449-463_(L452R/Y453F)-recognizing CD4^+^ Th cells. Spike_489-513(N501Y)_ peptide was also a promiscuously presented to HLA-DRB1*09:01 and DRB1*15:02. The T-cell responses specific to both peptides Spike_448-477_ and Spike_489-513_ were detected in PBMCs after vaccinations. In addition, we observed that the Spike_448-477_ peptide activated both CD8^+^ T-cells and CD4^+^ Th cells in individuals receiving mRNA vaccines. SARS-CoV-2 spike protein-derived peptides, Spike_448-477_ and Spike_489-513_, include several epitopes that are presented by multiple HLA-class II alleles to activate CD4^+^ Th cells, which are considered useful for monitoring the establishment of acquired immunity after vaccination.

## 1 Introduction

The current coronavirus disease 2019 (COVID-19) pandemic that is caused by the severe acute respiratory syndrome coronavirus 2 (SARS-CoV-2) poses a significant threat to public health [1]. In response to the outbreak of the disease, therapeutic drugs and vaccines have been developed to attenuate severe symptoms and to prevent SARS-CoV-2 infection, respectively [2]. The developed vaccines, which are based on the mRNA of the SARS-CoV-2 spike protein, have been shown to induce both cellular and humoral immunity against SARS-CoV-2 and to reduce mortality rates [[3], [4], [5]]. Peptide-based vaccine offers several benefits, such as cost-effectiveness and ease of synthesis [6]. In addition, peptides can be easily transported and stored at room temperature because they are stable and can be lyophilized. This makes the use of peptide vaccines particularly even in developing countries, where cold chain storage and transportation can be challenging.

Numerous studies have shown that T-cells play an important role in attenuating COVID-19, even in the absence of a humoral immune response [[7], [8], [9]]. Consequently, the induction of SARS-CoV-2 T-cell immunity has been a goal of vaccine development, with a particular focus on the maintenance of memory CD4^+^ helper T lymphocytes (Th cells). These CD4^+^ Th cells, which are involved in the differentiation of CD8^+^ T cells into effector cytotoxic T lymphocytes (CTLs) that kill infected cells before the virus particles are released into the environment, have been shown to prevent exacerbation of COVID-19 [10]. Because antigen recognition of T-cells is restricted to specific types of HLA alleles, it is important to identify peptides that possess a variety of epitopes that can be presented to HLA alleles when developing peptide-based vaccines.

In this study, we attempted to develop a peptide-based SARS-CoV-2 vaccine candidate that could activate both CD4^+^ Th cells and CTLs. Given that peptide-based vaccines that are capable of activating both CD4^+^ Th cells and CTLs effectively can induce cellular immunity [11,12], we focused on the Spike_448-477_ (NYNYLYRLFRKSNLKPFERDISTEIYQAGS) peptide, which includes the HLA-A*24:02-binding Spike_448-456_ (NYNYLYRLF) peptide [13]. In addition, we evaluated the immunogenicity of the Spike_489-513(N501Y)_ peptide (YFPLQSYGFQPTNGVGYQPYRVVVL), because a mutation of aspartic acid at position 501 has been identified in the several SARS-CoV-2 variants, such as alpha, beta, gamma, and omicrons. Collectively, we found that both peptides were promiscuous peptides; Spike_448-477_ peptide binds to HLA-DPB1*02:02, DRB4*01:03, and DRB1*08:02 and Spike_489-513(N501Y)_ peptide binds to HLA-DRB1*09:01 and DRB1*15:02. We also examined SARS-CoV-2-specific T-cell responses to these peptides in individuals that received the Pfizer-BioNTech mRNA vaccine. The findings showed that these peptides can be used to monitor SARS-CoV-2-specific acquired immunity.

## 2 Materials and methods

### 2.1 Cell lines

Lymphoblastoid cell lines (LCLs) a-d were generated from the peripheral blood mononuclear cells (PBMCs) of healthy volunteers by culturing the cells in the culture supernatant of the Epstein–Barr virus-producing B95-8 cell line [14]. L-cells (mouse fibroblasts) expressing transfected HLA class II molecules were kindly donated by Dr. R. Karr (Karr Pharma, St. Louis, MO) and Dr. T. Sasazuki (Kyushu University, Fukuoka, Japan). Although no authentication assay was performed for any of the cell lines used, all of the cell lines were meticulously cultured as recommended by the supplier and used within six months.

### 2.2 Clinical samples

PBMCs were obtained from healthy donors at Asahikawa Medical University. This study was approved by the Research Ethics Committee of Asahikawa Medical University (20144) and was performed in accordance with the Declaration of Helsinki. Written informed consent was obtained from all donors who provided peripheral blood samples.

### 2.3 *In vitro* generation of spike-reactive CD4^+^ Th cell lines

The procedure for the co-culture of CD4^+^ T-cells and autologous dendritic cells (DCs) was performed as described previously [15]. Briefly, monocytes and CD4^+^ T-cells were purified from PBMCs using MACS microbeads for CD14 and CD4, respectively (Miltenyi Biotech). Monocytes were differentiated into DCs by cultured with granulocyte macrophage colony-stimulating factor (GM-CSF) (50 ng/ml) and interleukin (IL)-4 (1000 IU/ml) in a 5% CO_2_ incubator for 7 d. The DCs were validated to express antigen presentation-associated molecules, such as HLA-class I (ABC), HLA-class II (DR), and CD80 before using them. The qualified DCs were collected and pulsed with the Spike_448-477_ or the Spike_489-513(N501Y)_ peptides (3 μg/ml for 3 h at room temperature) and then co-cultured with autologous CD4^+^ T-cells in 96-well flat-bottomed culture plates. Following a 7-day culture, the CD4^+^ T-cells were restimulated in individual microcultures with the cognate Spike peptide-pulsed, γ-irradiated, autologous PBMCs (3 μg/ml), followed by the addition of recombinant human IL-2 (10 IU/ml) 2 days later. To expand the Spike peptide-specific CD4^+^ Th cells for further analysis, the CD4^+^ T-cells were restimulated weekly using irradiated autologous PBMCs pulsed with the cognate Spike peptide (3 μg/ml). Production levels of IFN-γ in culture supernatants were determined using an ELISA kit (BD Pharmingen) according to the manufacturer's instructions. We measured absorption at 450 nm using a GloMax Discover Microplate Reader (Promega). AIM-V medium (Invitrogen) supplemented in 3% of human male AB serum (Innovative Research) was used as a complete culture medium for all experiments. All peptides used in this study were commercially synthesized by GenScript and provided from Medical & Biological Laboratories Co., Ltd. The HLA types of all healthy donors were identified through the PCR-rSSO method. All blood materials were acquired after informed consent was appropriately obtained.

### 2.4 Addressing spike-specific responses with established CD4^+^ Th cells

To evaluate specificity of the expanded CD4^+^ Th cells to the Spike peptids, autologous PBMCs were pulsed with various concentrations (0–30 μg/ml) of the Spike_448-477_ or the Spike_489-513(N501Y)_ peptides for 3 h, and subsequently washed with PBS twice in order to remove any free peptide. Then, the peptide-pulsed PBMCs were co-cultured with CD4^+^ Th cells in a 5% CO_2_ incubator for 24 h. To define the minimal epitopes of CD4^+^ Th cells by using truncated peptides, autologous PBMCs were pulsed with 3 μg/ml of each truncated peptide for 3 h, and subsequently washed with PBS twice in order to remove any free peptide. Then, the peptide-pulsed PBMCs were co-cultured with CD4^+^ Th cells in a 5% CO_2_ incubator for 24 h. Supernatants in each culture were collected to measure IFN-γ production levels using ELISA kits as mentioned above.

### 2.5 Addressing HLA-restriction of the spike-specific CD4^+^ Th cells

CD4^+^ Th cells (1–1.5 × 10^5^) were co-cultured with antigen-presenting cells (APCs) such as irradiated autologous PBMCs (1.5 × 10^5^), LCLs (3 × 10^4^), or HLA-DR-expressing L-cells (3 × 10^4^) in some experiments. To determine the HLA-class II restriction of the established CD4^+^ Th cells, APCs were pulsed with the Spike_448-477_ or the Spike_489-513(N501Y)_ peptides (3 μg/ml) for 3 h, followed by two washes with PBS to remove any unbound peptides. Then, the peptide-pulsed APCs were pre-treated with 10 μg/ml of anti-HLA-DP monoclonal antibody (mAb) BRAFB6 (Santa Cruz), anti-HLA-DQ mAb SPV-L3 (NOVUS Biologicals), anti-HLA-DR mAb L243 (IgG2a, prepared from the supernatants of hybridoma HB-55 obtained from ATCC), and anti-HLA-A/B/C (class I) mAb W6/32 (IgG2a; ATCC), which interfere with interaction between the peptide/HLA complex and the T-cell receptor, for 2 h, and co-cultured with CD4^+^ Th cell lines in a 5% CO_2_ incubator for 24 h. T-cell responses were evaluated by ELISA.

### 2.6 Evaluating frequency of spike-specific T-cells in a short-term culture system by ELISpot assay

PBMCs (1.5–2 × 10^6^) derived from healthy donors were stimulated with 1 μg/ml of the Spike_448-456_ (short peptide binding to HLA-A24), the Spike_448-456_, or the Spike_489-513_ peptides in the presence of IL-2 (20 IU/ml) in 24-well plates, as described previously [16]. Seven days after peptide stimulation, the PBMCs were washed with PBS twice and addressed their specificity to the Spike peptides by an enzyme-linked immune absorbent spot (ELISpot) assay. The washed PBMCs (4 × 10^4^) were cultured on a MAHAS4510 plate (Millipore) in the presence or absence of Spike peptides (1 μg/ml) for 24 h, and the number of IFN-γ-producing cells in the culture was measured using an ELISpot kit (Mabtech) according to the manufacturer's instructions. Plates were scanned with an automated ELISpot plate reader (Autoimmun Diagnostika GmbH). Spots were counted and analyzed using AID ELISPOT plate reader software (Autoimmun Diagnostika GmbH).

### 2.7 Major histocompatibility complex (MHC) tetramer production

The extracellular domains of HLA-DRA1*01:01 or HLA-DRB1*08:02 chains were expressed as fusions with the leucine zipper dimerization motifs of Fos or Jun. Constructs of the expression vector of HLA-DRA1*01:01 or HLA-DRB1*08:02 chains were tagged with a Fos-6xHis-tag or a Jun-BirA recognition sequence in frame at their C terminus ends, respectively. CHO–K1SV GSKO cells (Lonza) were transfected with these constructs by electroporation using a Neon Transfection system (Thermo Fisher Scientific), followed by a limiting dilution. Soluble recombinant HLA-DRA1*01:01 and HLA-DRB1*08:02 proteins were dimerized using a leucine zipper in the Fos-Jun interaction. The soluble HLA-DRA1*01:01/HLA-DRB1*08:02 heterodimer was purified by a Ni-sepharose excel column (GE Healthcare). After purification, the HLA-DRA1*01:01/HLA-DRB1*08:02 heterodimer was biotinylated using BirA according to the manufacturer's instructions (Avidity) before being conjugated with the synthetic Spike peptides, Spike_449-463(WT)_ (YNYLYRLFRKSNLKP) and Spike_449-463(L452R/Y453F)_ (YNYRFRLFRKSNLKP) and incubated at 37 °C for 24 h. The resulting monomeric HLA-DRA1*01:01/HLA-DRB1*08:02 heterodimer/peptide complexes were purified by monomeric avidin gel chromatography (Pierce), fractionated by liquid chromatography (AKTA25; GE Healthcare), and assessed for purity by HPLC (Waters). Tetrameric arrays of biotinylated HLA-DRA1*01:01/HLA-DRB1*08:02 heterodimer/peptide complexes were formed by the addition of phycoerythrin (PE)-labeled streptavidin (ProZyme) at a molar ratio of 4:1. All tetramers were synthesized and supplied by Medical & Biological Laboratories Co., Ltd. We validated whether the HLA-DRB1*08:02-restricted Spike_449-463_ peptide-specific CD4^+^ Th cells were detected by the tetramers. The CD4^+^ Th cells were treated with anti-Fc receptor mAb (Miltenyi Biotech) at room temperature for 5 min, and then treated with FITC-conjugated anti-CD3 mAb (HIT3a), PE/Cy7-conjugated anti-CD4 mAb (RPA-T4), APC/Cy7-conjugated anti-CD8 mAb (RPA-T8), and the PE-conjugated tetramers at 4 °C for 15 min. The antibodies were purchased from BioLegend. The CD4^+^ Th cells were washed with PBS supplemented in 0.5% of fetal bovine serum (Biowest) before analysis of their fluorescent levels, which were measured using a CytoFlex Flow Cytometer (Beckman Coulter). Live CD4^+^ Th cells were analyzed in based on forward and side scatter plots.

### 2.8 Statistical analysis

Statistical analysis was performed using GraphPad Prism 9.3.1 (GraphPad Software). Differences between two groups and among multiple groups were analyzed by using unpaired t tests and one-way ANOVA with Dunnett's multiple comparisons test, respectively. Data are presented as mean ± SD or SE, and P < 0.05 was considered statistically significant.

## 3 Results

### 3.1 Identification of SARS-CoV-2 spike protein-derived helper epitopes

**Fig. 1:**
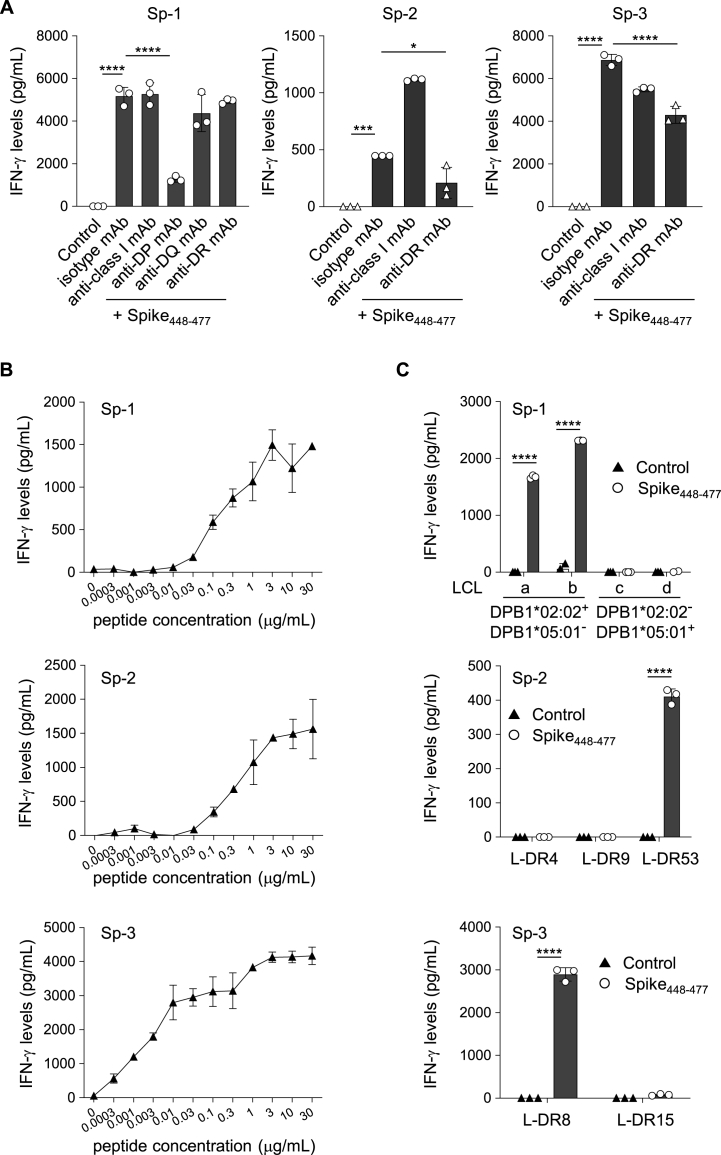
An immunogenic Spike_448-477_ peptide promiscuously presented to CD4^+^ Th cells. (A) Spike_448-477_-specific CD4^+^ Th cell lines (Sp-1, Sp-2, and Sp-3) were stimulated with autologous PBMCs pulsed with Spike_448-477_ peptide in the presence of isotype mAb, anti-HLA-class I mAb, anti-HLA-DP mAb, anti-HLA-DQ mAb, or anti-HLA-DR mAb. (B) Spike_448-477_-specific CD4^+^ Th cell lines (Sp-1, Sp-2, and Sp-3) were cocultured with autologous PBMCs in the presence of various concentrations (0–30 μg/ml) of Spike_448-477_ peptide. (C) HLA-restriction of the Sp-1 and Sp-2 and 3 was assessed by using Spike_448-477_ peptide-pulsed HLA-DPB1*02:02^+^DPB1*05:01^−^LCL lines (a and b) and HLA-DPB1*02:02^−^DPB1*05:01^+^LCL lines (c and d) (upper panel) and L-cells expressing individual HLA-DR allele (middle and lower panels), respectively. Supernatants were collected after 24 h for assessing IFN-γ production by ELISA in all experiments. Data are shown as mean ± SE. *, P < 0.05; ***, P < 0.001; ****, P < 0.0001; one-way ANOVA with interaction followed by Tukey's multiple comparisons test (A) and unpaired *t*-test (C). ND, not detected. Control represented co-culture with APCs and CD4^+^ Th cell lines without any peptides (A and C). Experiments were performed with at least 3 biological replicates and are representative of at least 2 independent experiments.

We evaluated whether the Spike_448-477_ (NYNYLYRLFRKSNLKPFERDISTEIYQAGS) peptide could activate CD4^+^ Th cells derived from the PBMCs of healthy donors. The CD4^+^ T-cells were stimulated with the peptide on autologous monocyte-derived DCs and restimulated weekly with peptide-pulsed, γ-irradiated, autologous PBMCs. As a result, we acquired three lines of Spike_448-477_-reactive CD4^+^ Th cells from two healthy donors (Sp-1 and Sp-2 from HLA-DP2/DP5 and DR4/DR53, and Sp-3 from HLA-DR8/DR15). To define the HLA-class II restriction, we evaluated the reactivity of these CD4^+^ Th cell lines to autologous PBMCs in the presence of the cognate peptide by using anti-HLA class I or HLA-class II mAbs. IFN-γ production by Sp-1 was inhibited by the antibody against HLA-DP, but not by antibodies against HLA class I, HLA-DQ, or HLA-DR. In addition, both Sp-2 and Sp-3 CD4^+^ Th cell lines responded to the peptide in an HLA-DR-restricted manner (Fig. 1A). These data indicated that the Spike_448-477_ peptide contains epitopes that are presented to several HLA-class II alleles, especially HLA-DP and DR. These three CD4^+^ Th cell lines responded to the peptide in a dose-dependent manner (Fig. 1B). Furthermore, we addressed the reactivity of these CD4^+^ Th cell lines using LCLs, or L-cells transfected with the HLA-DR allele gene, and found that Sp-1, Sp-2, and Sp-3 were restricted to DPB1*02:02, DR53, and DR8, respectively (Fig. 1C). These findings indicate that the Spike_448-477_ peptide is a promiscuous peptide with the potential to stimulate CD4^+^ Th cells at multiple HLA-class II alleles, such as DP2, DR53, and DR8.

### 3.2 Identification of the minimal epitopes in the Spike_448-477_ peptide

**Fig. 2:**
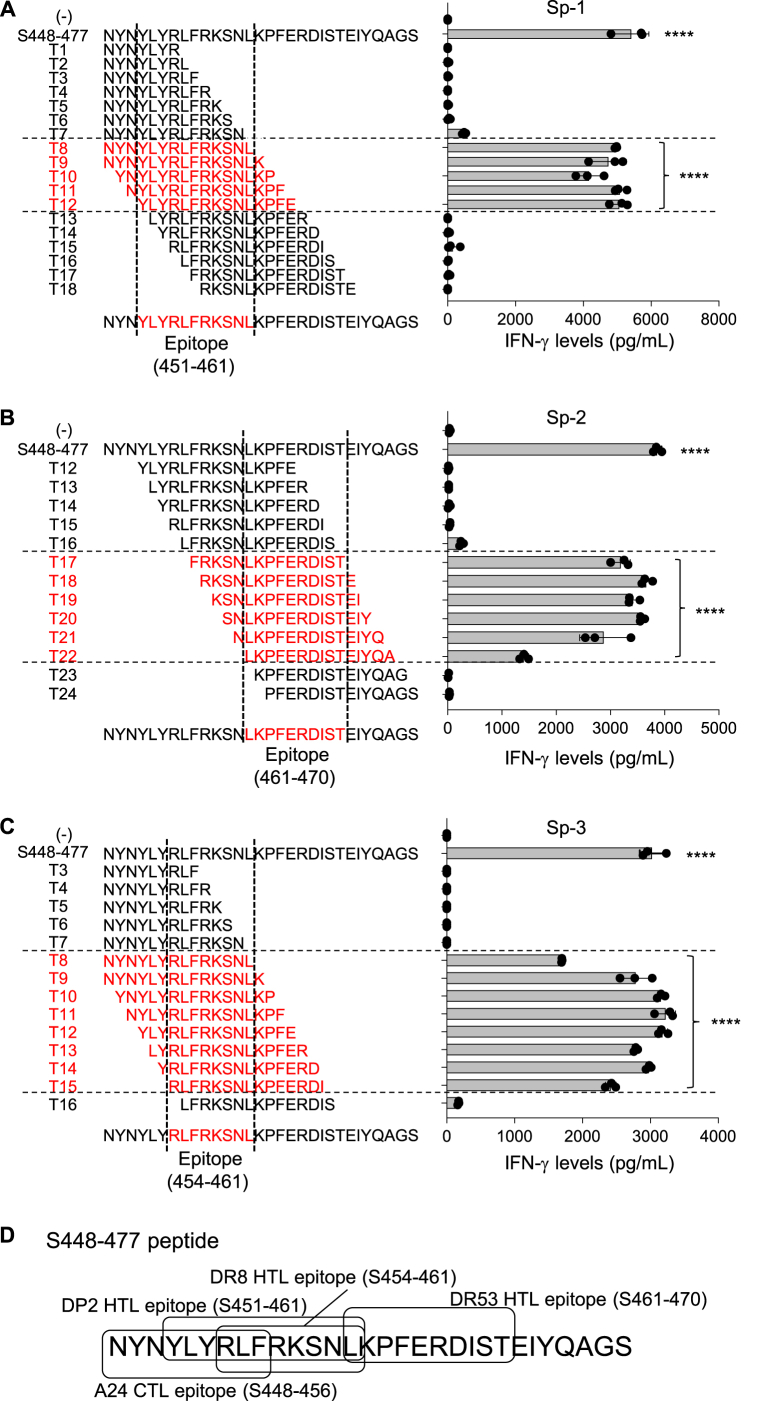
Defined minimal epitopes recognized by the Spike_448-477_-specific CD4^+^ Th cell lines. Sp-1 (A), Sp-2 (B), and Sp-3 (C) CD4^+^ Th cell lines were cultured with their autologous PBMCs in the presence of a series of 7–15-mer overlapping peptides encompassing Spike_448-477_ peptide (10 μg/ml). Reactive peptides and minimal epitopes of the three CD4^+^ Th cell lines were displayed with red font. Supernatants were collected after 24 h for assessing IFN-γ production by ELISA in all experiments. Data are shown as mean ± SE. ****, P < 0.0001; ordinary one-way ANOVA followed by Dunnett's multiple comparisons test (vs (−)). Experiments were performed with at least 3 biological replicates and are representative of at least 2 independent experiments.

To identify the minimal epitopes recognized by these three CD4^+^ Th cell lines, a series of 7–14-mer overlapping truncated peptides derived from the Spike_448-477_ peptide were tested for their ability to stimulate these CD4^+^ Th cell lines. The HLA-DPB1*02:02-restricted Sp1 responded to five truncated peptides, T8 to T12, suggesting that the minimal region was Spike_451-461_ (YLYRLFRKSNL) (Fig. 2A). Furthermore, the minimal epitopes of Sp-2 and Sp-3 were found to be Spike_461-470_ (LKPFERDIST) and Spike_454-461_ (RLFRKSNL), respectively (Fig. 2B and C). These epitopes are summarized with a CTL epitope in Fig. 2D.

### 3.3 Evaluation of reactivity to SARS-CoV-2 variants

**Fig. 3:**
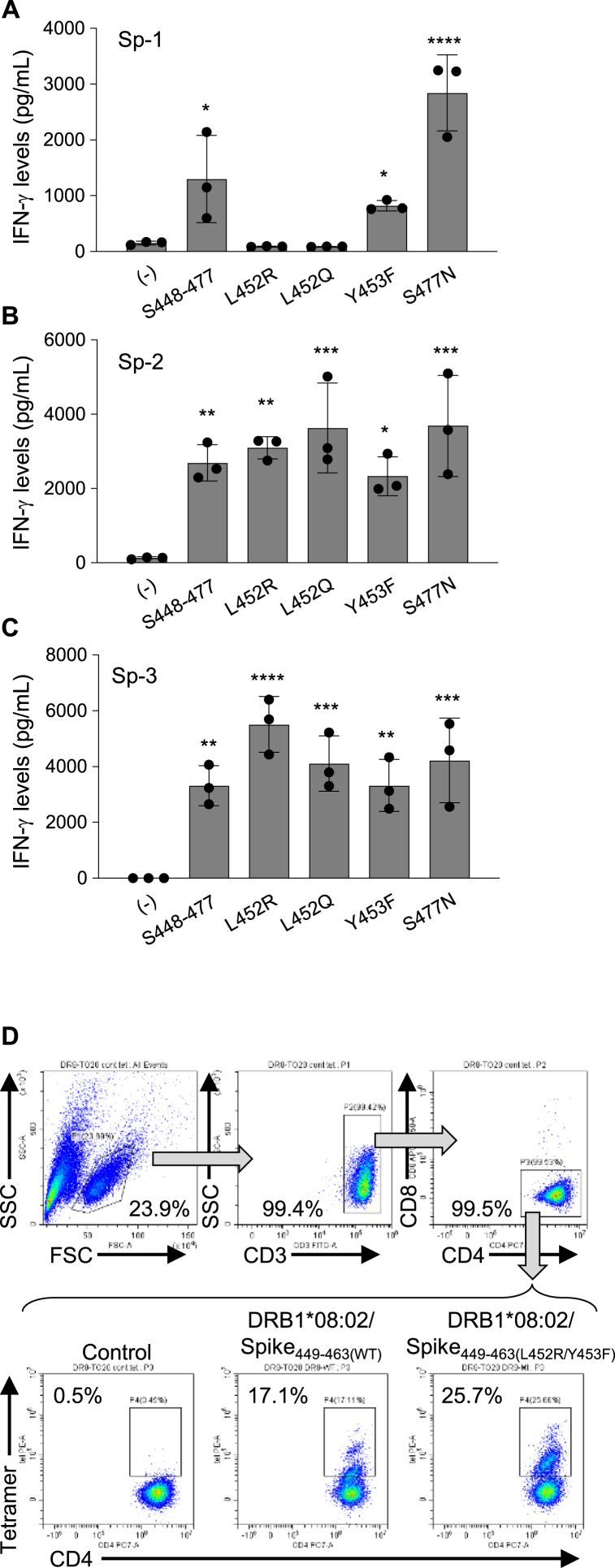
Reactivity of the Spike_448-477_-specific CD4^+^ Th cell lines to mutant peptides derived from some SARS-Cov-2 mutants. Spike_448-477_ peptide-derived mutant peptides were synthesized and added into the coculture of the Spike_448-477_-specific CD4^+^ Th cell lines with autologous PBMCs. Supernatants in the culture of the Sp-1 (A), Sp-2 (B), and Sp-3 (C) cell lines were collected after 24 h for assessing IFN-γ production by ELISA in all experiments, respectively. Data are shown as mean ± SE. **, P < 0.01; ***, P < 0.001; ****, P < 0.0001; ordinary one-way ANOVA followed by Dunnett's multiple comparisons test (vs (−)). Experiments were performed with at least 3 biological replicates and are representative of at least 2 independent experiments. PE-conjugated tetramers for DRB1*08:02/Spike_449-463 (WT)_ and DRB1*08:02/Spike_449-463 (L452R/Y453F)_ were synthesized and validated using the DRB1*08:02-restricted Sp-3 cell line with a flow cytometer (D). To gate CD4^+^ Th cell population, monoclonal antibodies against CD3, CD4, and CD8 were used.

SARS-CoV-2 frequently mutates in order to escape the immune system, and the position L452 is reported to have mutated in some SARS-CoV-2 variants, such as Delta, Kappa, Lambda, Epsilon, and the Omicrons [17,18]. Therefore, we tested whether these mutations affect the responses of Spike_448-477_ peptide-specific CD4^+^ Th cells. The CD4^+^ Th cell line Sp-1 did not recognize peptides with the L452R and L452Q mutations (Fig. 3A), while the other CD4^+^ Th cell lines Sp-2 and Sp-3 were not affected by any mutant peptides (Fig. 3B and C). The Y453F, which has been found in both human and mink in Denmark [19], and the S477 N, which is shared by the Iota and Omicron variants [17,18], were used as control peptides and induced IFN-γ production from all the CD4^+^ Th cell lines tested (Fig. 3A–C). These findings suggested that these HLA-DR53- or DR8-restricted CD4^+^ Th cells could broadly respond to the SARS-CoV-2 variants reported in the literature. Thus, we generated two tetramers to detect HLA-DRB1*08:02/Spike_449-463_- and Spike_449-463_(L452R/Y453F)-recognizing CD4^+^ Th cells and found that both tetramers detected the Sp-3 cell line (Fig. 3D).

### 3.4 Identification of the critical amino acids in the epitope sequences

**Fig. 4:**
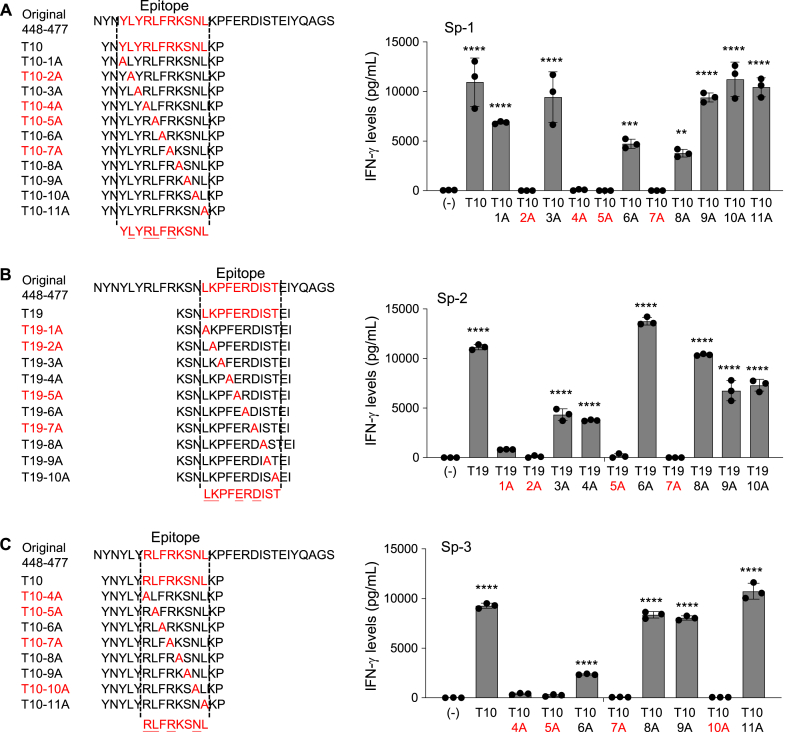
Responses of the Spike_448-477_-specific CD4^+^ Th cell lines to alanine-scan mutant peptides. Spike_448-477_ peptide-derived 15-mer alanine-scan mutant peptides with their epitopes were designed as shown in left panel. Sp-1 (A), Sp-2 (B), and Sp-3 (C) Th cell lines were cultured with their autologous PBMCs in the presence of the mutant peptides (10 μg/ml). Supernatants were collected after 24 h for assessing IFN-γ production by ELISA in all experiments. Data are shown as mean ± SE. *, P < 0.05; **, P < 0.01; ***, P < 0.001; ****, P < 0.0001; ordinary one-way ANOVA followed by Dunnett's multiple comparisons test (vs (−)). Experiments were performed with at least 3 biological replicates and are representative of at least 2 independent experiments.

To evaluate which position of amino acid is important for the responses of these CD4^+^ Th cell lines, we used alanine-substituted peptides derived from the Spike_448-477_ peptide. The results showed that T10-2A, 4A, 5A, and 7A peptides decreased the responses of the Sp-1 cell line, suggesting that L452, R454, L455, and R457 are critical positions for the Sp-1 cell line (Fig. 4A). L461, K462, F465, and D467 and R454, L455, R457, and N460 are critical positions for the recognition of the Sp-2 and the Sp-3 cell lines, respectively (Fig. 4B and C). These findings were consistent with the above results and demonstrated that the Sp-1 cell line only failed to respond to L452R and L452Q, but not Y453F and S477 N (Fig. 3A).

### 3.5 Identification of determinant amino acids in the epitope sequences

**Fig. 5:**
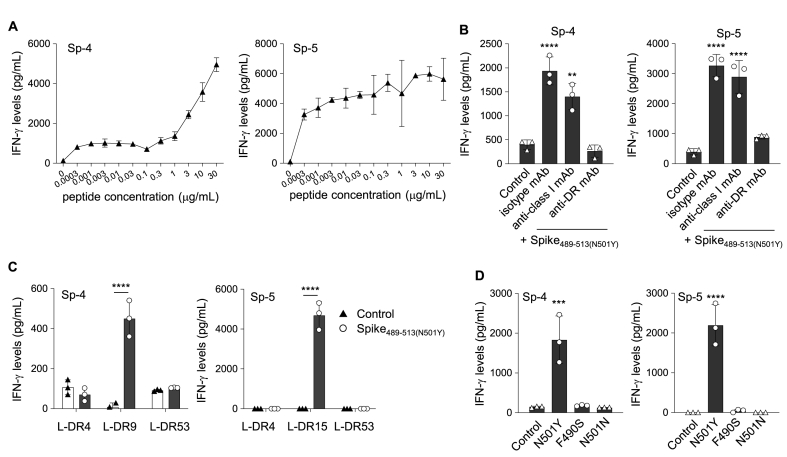
Promiscuously presented Spike_489-513 (N501Y)_ peptide to CD4^+^ Th cells. (A) Spike_489-513(N501Y)_-specific CD4^+^ Th cell lines (Sp-4 and -5) were cocultured with autologous PBMCs in the presence of various concentrations (0–30 μg/ml) of Spike_489-513 (N501Y)_ peptide. (B) Spike_489-513 (N501Y)_-specific CD4^+^ Th cell lines Sp-4 (left) and −5 (right) were stimulated with autologous PBMCs pulsed with Spike_489-513 (N501Y)_ peptide in the presence of isotype mAb, anti-HLA-class I mAb, or anti-HLA-DR mAb. (C) HLA-restriction of the Sp-4 (left) and −5 (right) was assessed by using Spike_489-513 (N501Y)_ peptide-pulsed L-cells expressing individual HLA-DR allele, respectively. (D) The Sp-4 (upper) and −5 (lower) lines were cultured with their autologous PBMCs in the presence of the mutant peptides (10 μg/ml). Supernatants were collected after 24–48 h for assessing IFN-γ production by ELISA in all experiments. Data are shown as mean ± SE. **, P < 0.01; ***, P < 0.001; ****, P < 0.0001; ordinary one-way ANOVA followed by Dunnett's multiple comparisons test (vs Control) (B) and (D) and unpaired *t*-test (C). ND, not detected. Control represented co-culture with autologous PBMCs and CD4^+^ Th cell lines without any peptides. Experiments were performed with at least 3 biological replicates and are representative of at least 2 independent experiments.

Next, we evaluated whether the Spike_489-513(N501Y)_ peptide (YFPLQSYGFQPTNGVGYQPYRVVVL) can induce CD4^+^ Th cell responses, because the N501Y mutation is also frequently shared by the Alpha, Beta, Gamma, and Omicron SARS-CoV-2 variants [19]. CD4^+^ T-cells were purified from the PBMCs of healthy donors and stimulated with the Spike_489-513(N501Y)_ peptides, as described above. From two healthy donors, we generated two Spike_489-513(N501Y)_-specific CD4^+^ Th cell lines (Sp-4 from HLA-DR4/DR9/DR53 and Sp-5 from HLA-DR9/DR15), which responded to the peptide in a dose-dependent manner (Fig. 5A). IFN-γ production by both CD4^+^ Th cell lines was inhibited by the HLA-DR mAb, but not the HLA class I mAb, indicating that these CD4^+^ Th cell lines were restricted to HLA-DR (Fig. 5B). Moreover, we found that the Sp-4 and the Sp-5 cell lines responded to the Spike_489-513(N501Y)_ peptide in HLA-DR9 and DR15-expressing L-cells, respectively (Fig. 5C). These findings suggest that the Spike_489-513(N501Y)_ peptide is also a promiscuous peptide. Given that the F490S mutation was reported within the Spike_489-513(N501Y)_ peptide of the Lambda strain, we addressed whether the mutation affected their responses. The findings showed that the CD4^+^ Th cell lines failed to recognize both the F490S and N501 intact peptides.

### 3.6 Monitoring immunity against SARS-CoV-2 after mRNA vaccination

**Fig. 6:**
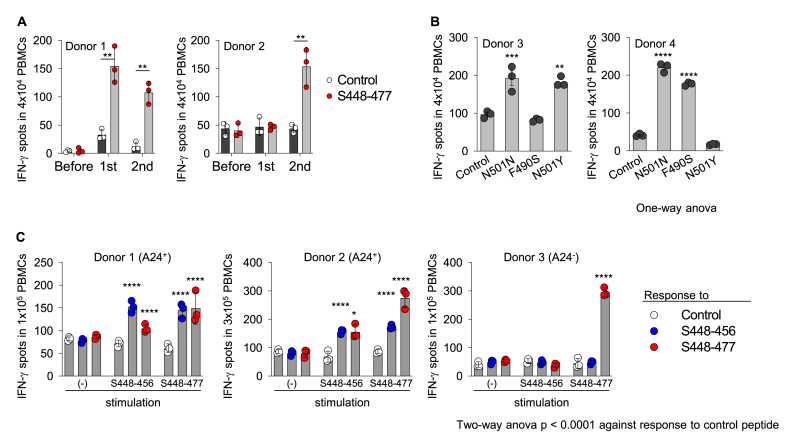
Evaluating utility of the Spike_448-477_ and Spike_489-513 (WT/N501Y)_ peptides as a tool for monitoring T-cell based immunity in the vaccinated subjects. (A) PBMCs were collected from two healthy donors before and after SARS-Cov-2 mRNA vaccinations and stocked in the refrigerator. Thewed PBMCs were stimulated with Spike_448-477_ peptide (1 μg/ml) in the presence of IL-2 (20 IU/ml). Seven days later, PBMCs were washed with PBS twice and then stimulated with Spike_448-477_ peptide (1 μg/ml) for 24 h. (B) PBMCs were collected from two healthy donors, who received the SARS-Cov-2 mRNA vaccines and stimulated with Spike_489-513 (WT)_ peptides (1 μg/ml) in the presence of IL-2 (20 IU/ml). Seven days later, PBMCs were washed with PBS twice and then stimulated with irrelevant (Control), Spike_489-513 (WT)_, Spike_489-513 (N501Y)_, or Spike_489-513 (F490S)_ peptides (1 μg/ml) for 24 h. (C) Freshly isolated PBMCs were collected from three donors positive or negative for HLA-A24 and cultured with or without the Spike_448-477_ peptide and the A24-binding CTL peptide Spike_448-456_ in the presence of IL-2 (20 IU/ml). for 7 days. Seven days later, PBMCs were washed with PBS twice and then stimulated with Spike_448-456_ or Spike_448-477_ peptides for 24 h. IFN-γ-producing cell numbers were measured by ELISPOT assay. Data are shown as mean ± SE. **, P < 0.01; ***, P < 0.001; ****, P < 0.0001; multiple unpaired *t*-test (A), ordinary one-way ANOVA followed by Dunnett's multiple comparisons test (vs Control) (B), and two-way ANOVA (vs Control) (C). Control represented that PBMCs were cultured without any peptides in ELISpot assay. ND, not detected. Experiments were performed with at least 3 biological replicates.

Millions of people have received mRNA vaccines based on SARS-CoV-2 spike proteins to prevent them from contracting COVID-19. We therefore addressed whether our identified peptides are useful for monitoring T-cell-based immune responses in vaccinated donors by evaluating the frequencies of peptide-specific T-cells using an ELISpot assay. In donor 1, Spike_448-477_ peptide-specific T-cell responses were detected immediately after the first vaccination. On the other hand, no T-cell response was detected in donor 2 until after the second vaccination (Fig. 6A). In the case of the Spike_489-513_ peptide, donor 3 showed T-cell responses against not only the Spike_489-513_ peptide (N501 N), but also the N501Y peptide. In contrast, the T-cells of donor 4 recognized the F490S peptide, but not the N501Y peptide (Fig. 6B). These findings suggested that our identified peptides, Spike_448-477_ and Spike_489-513_, were useful for monitoring cellular immunity against mutants of SARS-CoV-2. We addressed whether the Spike_448-477_ peptide activates both CTLs and CD4^+^ Th cells. Freshly isolated PBMCs were cultured in the absence or presence of the Spike_448-477_ peptide or the HLA-A24-binding CTL peptide Spike_448-456_ for 7 days. Then the PBMCs were washed with PBS twice and addressed their specific IFN-γ production to each peptide by ELISpot assay. As the results, the pre-stimulated PBMCs with the Spike_448-477_ peptide showed an equal response to the CTL peptide Spike_448-456_ in donors positive for HLA-A24 as the pre-stimulated PBMCs with the CTL peptide did (Fig. 6C). These findings suggest that the Spike_448-477_ peptide activates both CTLs and CD4^+^ Th cells.

## 4 Discussion

Here, we have demonstrated that the SARS-CoV-2 spike protein-derived peptide, Spike_448-477_ activates both CTLs and CD4^+^ Th cells. The CD4^+^ Th cell responses were restricted to HLA-DPB1*02:02, DRB1*08:02, or DR53. In addition, we have presented that the Spike_489-513(N501Y)_ peptide elicits CD4^+^ Th cell responses in a manner depending on HLA-DRB1*09:01 and DRB1*15:02. According to literature sources, the prevalence of HLA-DR8, which includes DRB1*08:02, HLA-DR9, which includes DRB1*09:01, and HLA-DR2, which includes DRB1*15:02 are shared to 15.1%, 11.9%, and 20.5%, respectively. HLA-DR53 is shared among individuals who have HLA-DR4 (24.4%), DR7 (14.0%), and DR9 (11.9%) [20]. Meanwhile, the prevalence of HLA-DPB1*02:02 is shared to only 1.1% in the world [21]. These peptides have induced SARS-CoV-2-specific T-cell responses in individuals that received the SARS-CoV-2 mRNA vaccine, and therefore may be useful for monitoring the establishment of acquired immunity after vaccination although the peptide alone cannot provide coverage for all individuals worldwide due to the limitation of the HLA restriction.

In addition to the mRNA-based vaccines, peptide-based vaccines have been developed for SARS-CoV-2 [22,23]. Although the peptide-based vaccines induce T-cell-based immune responses, but not humoral responses, that attenuate SARS-CoV-2 infection, increased frequency of memory T-cells specific for SARS-CoV-2 is also important for preventing infected hosts from exacerbating the disease [7]. The advantage of peptide-based vaccines is that they target cell surface molecules as well as intracellular molecules, because SARS-CoV-2-specific CD4^+^ Th cells and CTLs recognize peptides, which are processed intracellularly and then presented by MHC on the surface of antigen-presenting cells or infected cells. Therefore, SARS-CoV-2-derived proteins, such as ORF1ab and nucleocapsid phosphoprotein, which are localized intracellularly, could be also useful targets for peptide-based vaccines. Indeed, Heitmann et al. identified several CD4^+^ Th cell-stimulating peptides derived from spike, nucleocapsid, membrane, envelope, and ORF8 proteins. They showed that a vaccine composed of these peptides induced broad and potent T-cell responses with a favorable safety profile in a clinical setting [24]. In addition, Pardieck et al. demonstrated that a single CTL epitope protected against SARS-CoV-2 infection in a murine model [25]. While they did not investigate whether activation of CD4^+^ Th cells promotes CTL responses and extends survival of SARS-CoV-2 challenged mice, simultaneous activations of CD4^+^ Th cells and CTLs would boost vaccine efficacy because CD4^+^ Th cells enhance CTL activation in several ways including maturation of antigen-presenting cells such as DCs via CD40^−^CD154 interaction and production of robust IL-2 [26,27]. These literatures suggest that peptide-based vaccine would be one of the promising strategies for protecting against SARS-CoV-2.

Because we focused on only the spike protein as an antigen in the present study, future studies should extend our detailed epitope analysis to other molecules. Given that there is no localization limit with peptide-based vaccines, it might be a good strategy to select a molecule that plays a critical role in viral replication as a target antigen to prevent viruses from escaping the immune system. From this perspective, several reports have been published about peptide-based tumor vaccines targeting pro-tumor molecules in the field of cancer immunotherapy. Survivin-2B belongs to the inhibitor of apoptosis protein family and has been reported to be immunogenic [28]. We previously reported that survivin 2B-targeting vaccines effectively induced survivin 2B-specific T-cell responses in clinical settings [29,30].

Several SARS-CoV-2 variants have been identified during this global pandemic. The fifth variants of concern, Omicron BA.1 and BA.2, were reported to be an immune escape variant [31]. Furthermore, Emmelot et al. showed that Omicron BA.1 mutations resulted in decreased T-cell responses in individuals with immunity against the ancestral SARS-CoV-2 [32]. Although 20 CD4^+^ Th cell epitopes of the spike-protein-harboring Omicron BA.1 mutations were selected in the study [34], our defined Spike_448-477_ peptide was not included in the peptides. However, a mutation was observed at position 452 of the spike protein where a leucine was substituted with an arginine in the variants Delta, Kappa, Epsilon, and Omicron (BA2.11, BA4, and BA5), or a glutamine in the variants Lambda and Omicron (BA2.12.1). Given that L452 was found to be important for the peptide recognition by the HLA-DPB1*02:02-restricted CD4^+^ Th cells, individuals who possess the allele would have a reduced CD4^+^ Th cell responses against these cognate variants. In contrast, we showed that HLA-DR53 or DR8-restricted SARS-CoV-2-specific CD4^+^ Th cell responses were not affected by these mutations because the amino acids that are critical to recognition were not located at the position 452. These findings suggest that individuals possessing these HLA-alleles could have resistance to these mutated SARS-CoV-2 variants. Interestingly, reduced responses to the F490S and N501Y mutations in the Spike_489-513_ peptide were observed in the PBMCs of donors that received the Pfizer-BioNTech vaccine. These findings indicate that there are multiple epitopes contained in the peptide. Indeed, we acquired two lines of Spike_489-513(N501Y)_ peptide-specific CD4^+^ Th cells when we stimulated purified CD4^+^ T-cells from two individuals that were neither vaccinated nor infected. These Spike_489-513(N501Y)_ peptide-specific CD4^+^ Th cell lines were restricted to HLA-DRB1*09:01 and 15:02, respectively, and did not show any cross-reactivity to the wild-type peptide. Collectively, the Spike_489-513_ peptide is presented promiscuously by several HLA alleles as well as the Spike_448-477_ peptide, although their minimal epitopes remain to be elucidated.

## Ethics approval and consent to participate

The research protocols have been approved by the Research Ethics Committee of Asahikawa Medical University and performed in accordance with the Declaration of Helsinki. All participants have been informed of the potential risks and benefits and each donor has signed the informed consent form.

## Consent for publication

Not applicable.

## Author contribution statement

**Yuki Yajima**: Performed the experiments; Analyzed and interpreted the data. **Akemi Kosaka**: Performed the experiments; Analyzed and interpreted the data; Contributed reagents, materials, analysis tools or data. **Takayuki Ohkuri**: Conceived and designed the experiments; Performed the experiments; Analyzed and interpreted the data; Contributed reagents, materials, analysis tools or data; Wrote the paper. **Yoshihiko Hirohashi**: **Toshihiro Nagato**: Analyzed and interpreted the data. **Dongliang Li**: Performed the experiments; Analyzed and interpreted the data; Contributed reagents, materials, analysis tools or data; Wrote the paper. **Takeshi Nagasaki**: **Toshihiko Torigoe**: Conceived and designed the experiments; Analyzed and interpreted the data. **Hiroya Kobayashi**: Conceived and designed the experiments; Performed the experiments; Analyzed and interpreted the data; Wrote the paper.

## Data availability statement

Data will be made available on request.

## Declaration of competing interest

The authors declare the following financial interests/personal relationships which may be considered as potential competing interests:Hiroya Kobayashi has been provided with all peptides and tetramers from Medical & Biological Laboratories Co., Ltd.Co-authors currently employed by Medical & Biological Laboratories Co., Ltd.-Li D, and Nagasaki T.

## List of abbreviations

COVID-19: coronavirus disease 2019
SARS-CoV-2: severe acute respiratory syndrome coronavirus 2
Th cells: helper T lymphocytes
CTLs: cytotoxic T lymphocytes
LCLs: lymphoblastoid cell lines
PBMCs: peripheral blood mononuclear cells
DCs: dendritic cells
GM-CSF: granulocyte macrophage colony-stimulating factor
mAb: monoclonal antibody
PE: phycoerythrin
ORF: open reading frame
